# Adult-onset diagnosis of interrupted aortic arch presenting with uncontrolled hypertension

**DOI:** 10.1093/ehjcr/ytag465

**Published:** 2026-06-23

**Authors:** Robert Zheng, Shusuke Yagi, Masataka Sata

**Affiliations:** Department of Cardiovascular Medicine, Tokushima University Hospital, 3-18-15 Kuramoto-cho, Tokushima 770-8503, Japan; Department of Community and Family Medicine, Tokushima University Graduate School of Biomedical Sciences, 3-18-15 Kuramoto-cho, Tokushima 770-8503, Japan; Department of Cardiovascular Medicine, Tokushima University Hospital, 3-18-15 Kuramoto-cho, Tokushima 770-8503, Japan

**Keywords:** Adult congenital arterial disease, Secondary arterial hypertension, Collateral circulation

A 67-year-old woman presented with uncontrolled hypertension (170/80 mmHg) despite receiving antihypertensive therapy for 8 years. She had no history of intermittent claudication, and no cardiac murmur was detected on physical examination. Bilateral dorsalis pedis pulses were faintly palpable, and the ankle-brachial index was 0.5 bilaterally. Contrast-enhanced computed tomography (CT) and aortography revealed a type A interrupted aortic arch (IAA) distal to the dilated left subclavian artery, (*Panel A*, arrow).

**Figure:**
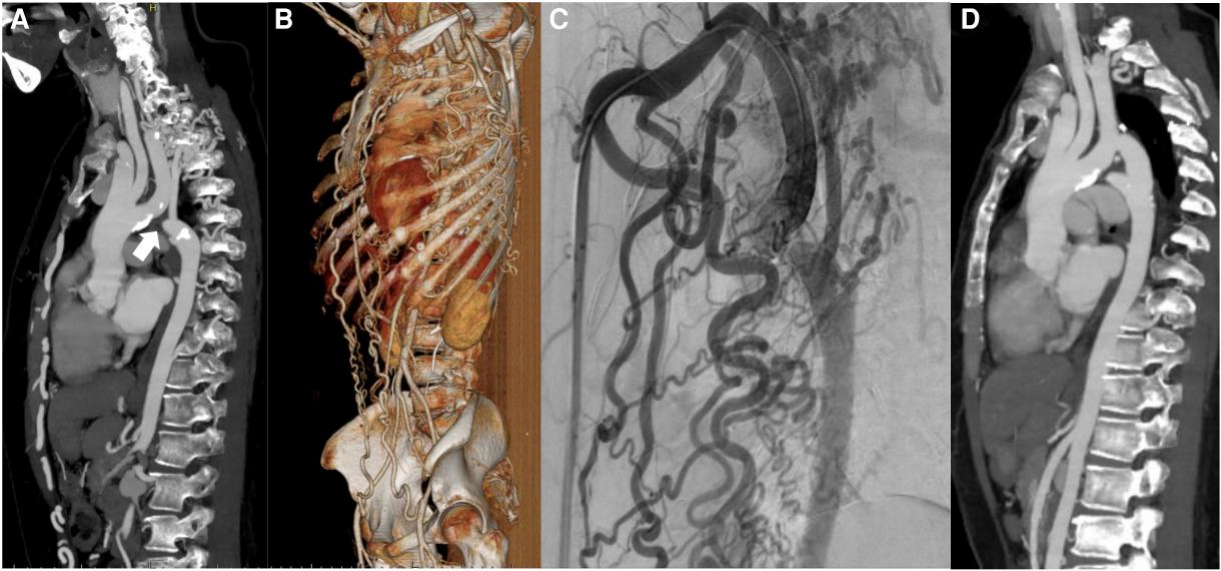


Blood supply to the lower extremities was maintained through collateral channels originating from the bilateral internal thoracic arteries (*Panel B*). Aortography demonstrated opacification of the distal aorta without contrast delay, owing to abundant collateral blood flow from the left subclavian artery (*Panel C*). Transoesophageal echocardiography revealed no patent ductus arteriosus or other congenital anomalies.

Plasma renin activity was elevated (44 ng/ml/h), whereas the plasma aldosterone concentration remained within the normal range (88 ng/ml). Electrolyte levels, renal function, and other endocrinological evaluations were unremarkable. These findings suggested that the patient's secondary proximal hypertension was attributable to IAA. Increased vascular resistance proximal to the interruption results in chronic pressure overload in the upper body, while reduced renal perfusion may activate the renin–angiotensin–aldosterone system, contributing to hypertension.

A left subclavian artery-to-descending aorta bypass graft was performed without complications. Blood pressure normalized to 110/64 mmHg immediately after surgery and remained well controlled during 1 year of follow-up. Contrast-enhanced CT demonstrated resolution of the abundant collateral flow from the bilateral subclavian arteries (*Panel D*).

Typically, the onset of pulmonary respiration in term infants leads to ductus arteriosus closure within 2–3 weeks from birth.^1^ If adequate collateral circulation does not develop, peripheral perfusion is compromised, causing ductal shock from lactic acidosis and thus, a poor prognosis.^2^ In this case, gradual ductus arteriosus closure provided enough time for developing collateral circulation. The patient remained free of major complications until late adulthood and represents one of the oldest reported cases of IAA diagnosed in adulthood.^2^ IAA might be the underlying cause when encountering unexplained and uncontrolled hypertension. IAA, although extremely rare in adults, should be considered in patients with long-standing unexplained hypertension, particularly when blood pressure discrepancies or diminished lower-extremity pulses are present.

## Data Availability

The data underlying this article will be shared on reasonable request to the corresponding author.

## Notes

**Consent:** The authors confirm that a written consent for submission and publication of this case report, including images and text, was obtained from the patient in line with the COPE guidelines.
